# Supplementary material for: Dislocation avalanches are like earthquakes on the micron scale
Source: Nat Commun. 2022 Apr 13;13:1975. doi: 10.1038/s41467-022-29044-7 (PMC9007997; doi:10.1038/s41467-022-29044-7)
Supplement: Supplementary file 1 — Supplementary Information [file 41467_2022_29044_MOESM1_ESM.pdf]

# SUPPLEMENTARY INFORMATION
# Dislocation avalanches are like earthquakes on the micron scale

Péter Dusán Ispánovity[a,*], Dávid Ugi[a,*], Gábor Péterffy[a], Michal Knapek[b], Szilvia Kalácska[a,c], Dániel Tüzes[a], Zoltán Dankházi[a], Kristián Máthis[b], František Chmelík[b], István Groma[a]

[a] *Eötvös Loránd University, Department of Materials Physics, 1117 Budapest, Pázmány Péter sétany 1/a. Hungary*
[b] *Charles University, Faculty of Mathematics and Physics, Department of Physics of Materials, 121 16 Prague 2, Ke Karlovu 5, Czech Republic*
[c] *Mines Saint-Etienne, Univ Lyon, CNRS, UMR 5307 LGF, Centre SMS, 158 cours Fauriel 42023 Saint-Étienne, France*

---

*Keywords:* Crystal plasticity, dislocation avalanche, strain burst, micromechanics, acoustic emission

---

*Corresponding author

*Email addresses:* peter.ispanovity@ttk.elte.hu (Péter Dusán Ispánovity), david.ugi@ttk.elte.hu (Dávid Ugi)

## Supplementary Figures

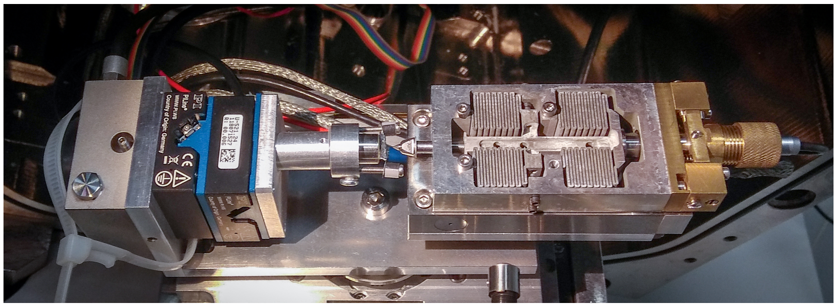

Supplementary Figure 1: **In-house developed *in situ* nanoindentation device.**

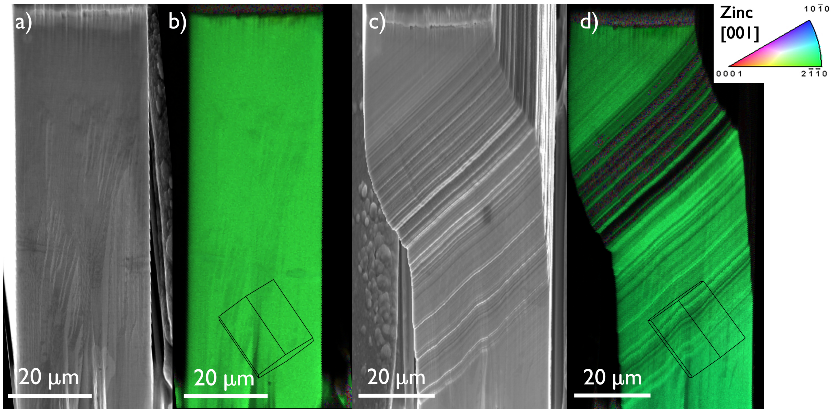


Supplementary Figure 2: **SEM imaging of the micropillars.** **a**, **c**, Secondary electron image of the same pillar in a tilt-corrected (70°) view before and after deformation. Note the uni-directional parallel slip bands in the deformed pillar. **b**, **d**, EBSD orientation map measured before and after compression of a Zn micropillar. The uniform color confirms single crystal structure both before and after the deformation. The orientation of the unit cell is also shown proving that the slip bands are parallel with the basal plane of the crystal.

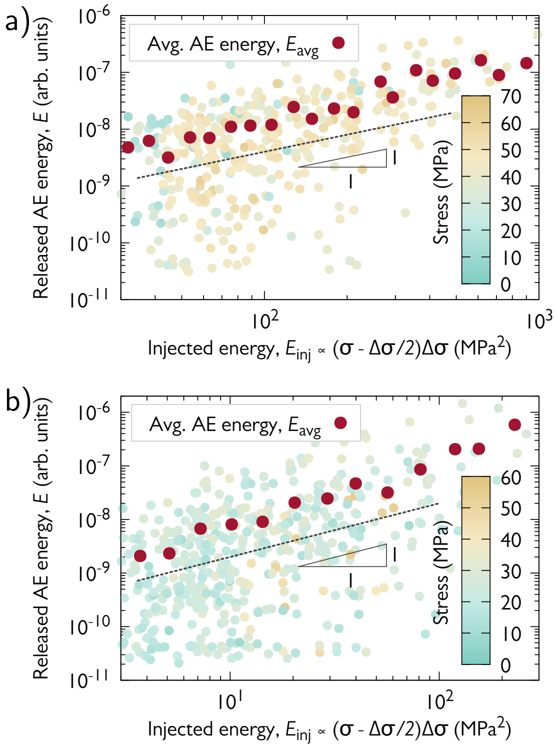


Supplementary Figure 3: **Correlation between stress drops and released AE energy.** **a**, Equivalent figure to that of Fig. 2c for $d = 8$ µm micropillars. **b**, Equivalent figure to that of Fig. 2c for $d = 16$ µm micropillars.

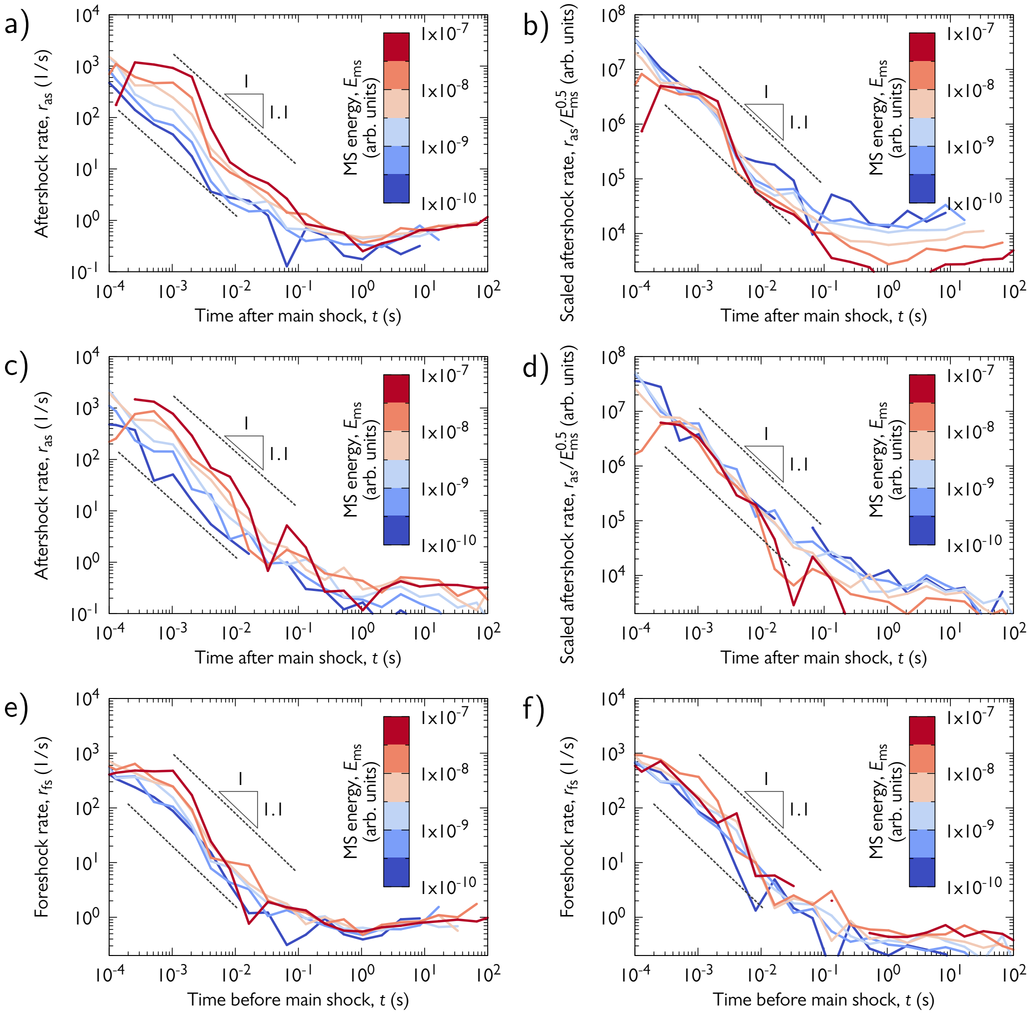


Supplementary Figure 4: **Aftershock and foreshock rates for $d = 8$ µm and $d = 16$ µm micropillars.** **a**, Aftershock rates $r_{as}$ after main shocks of various energies $E_{ms}$ for $d = 8$ µm micropillars. **b**, Aftershock rates $r_{as}$ of panel a) scaled with $E_{ms}^{0.5}$ for $d = 8$ µm micropillars. **c**, Aftershock rates $r_{as}$ after main shocks of various energies $E_{ms}$ for $d = 16$ µm micropillars. **d**, Aftershock rates $r_{as}$ of panel a) scaled with $E_{ms}^{0.5}$ for $d = 16$ µm micropillars. **e**, Foreshock rates $r_{fs}$ before main shocks of various energies $E_{ms}$ for $d = 8$ µm micropillars. **f**, Foreshock rates $r_{fs}$ before main shocks of various energies $E_{ms}$ for $d = 16$ µm micropillars.

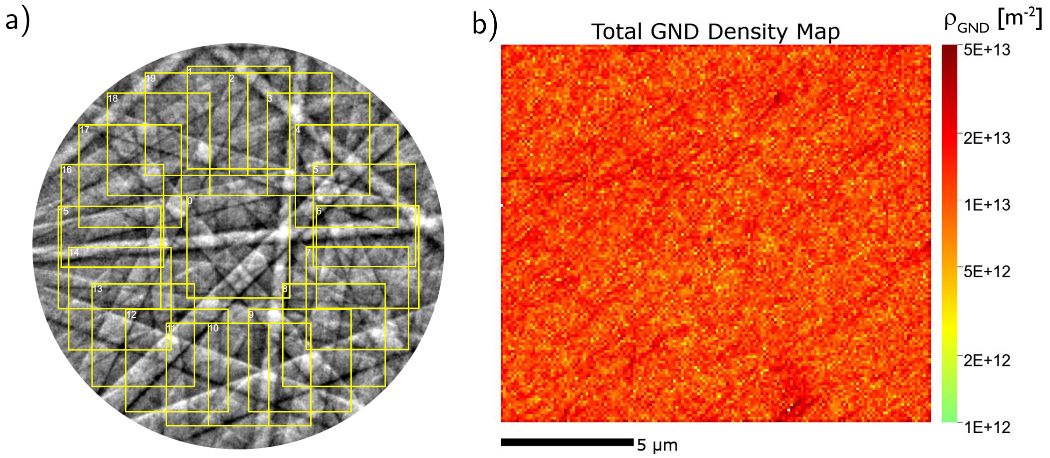


Supplementary Figure 5: **a,** A typical Kikuchi pattern collected from the sample used for the HR-EBSD evaluation. 20 regions of interest are marked with yellow squares. **b,** The resulting GND density map. Black point (in the middle) marks the reference pixel.

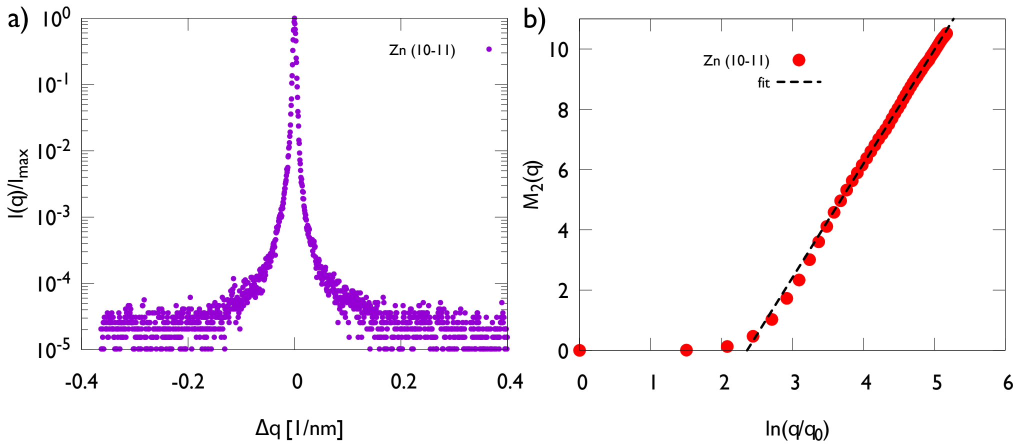


Supplementary Figure 6: **X-ray diffraction measurements on the original bulk Zn sample.** **a**, The measured X-ray line profile of the $(10\bar{1}1)$ reflection of the Zn single crystal. **b**, Second restricted moment $M_2$ as a function of $\ln q/q_0$, with $q_0 = 1.0$ 1/nm. Dislocation density can be obtained from the linear fit from Eq. (1).

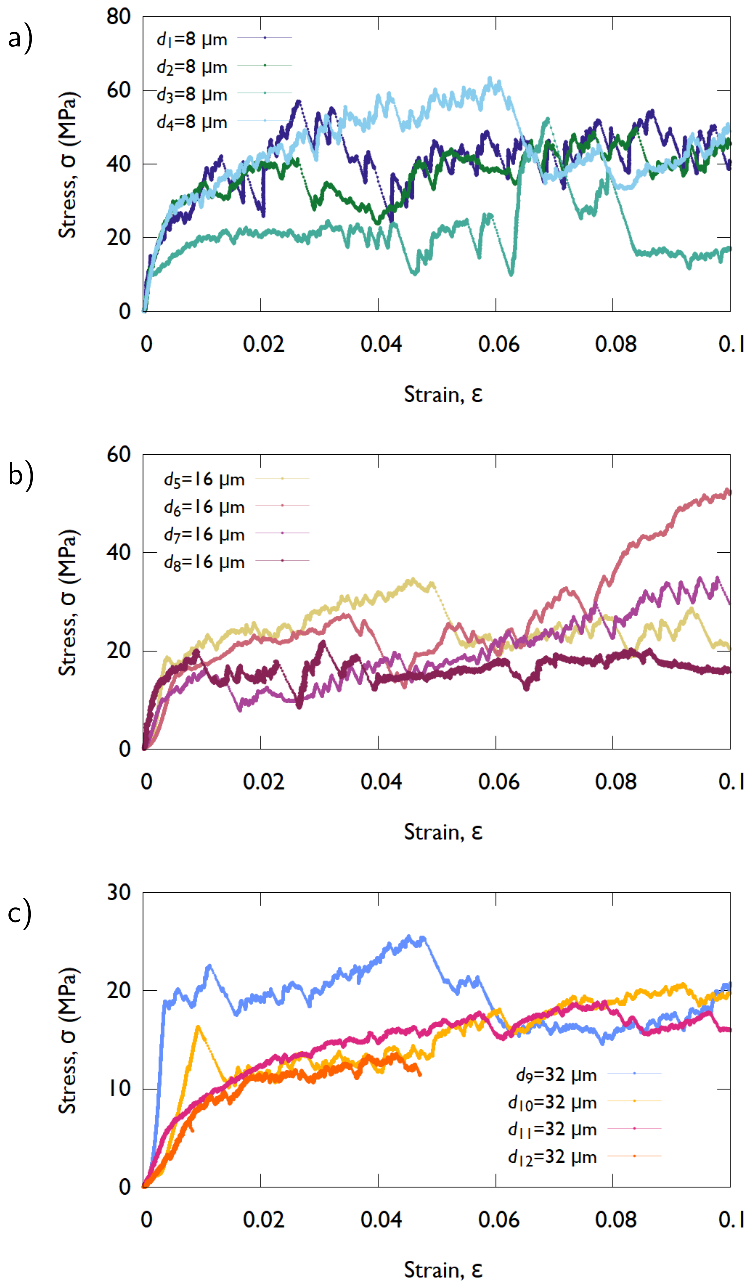


Supplementary Figure 7: **Exemplary stress-strain curves of micropillars of various sizes.** **a**, $d = 8$ µm, **b**, $d = 16$ µm and **c**, $d = 32$ µm pillars.

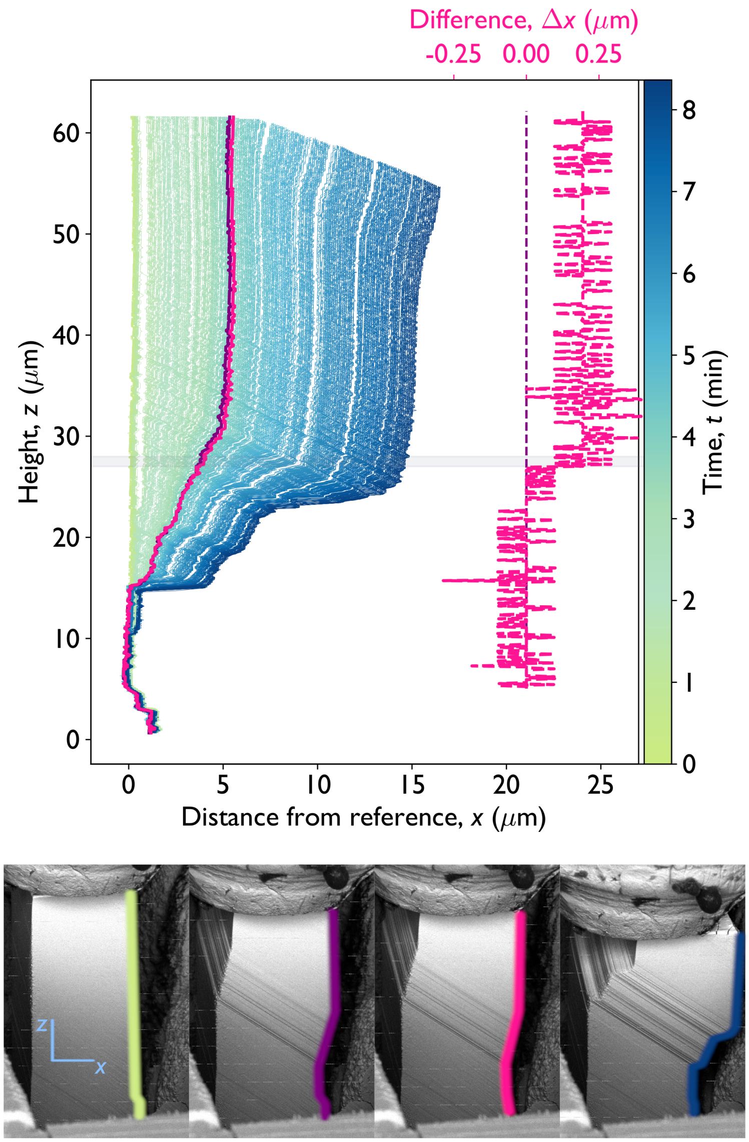


Supplementary Figure 8: **Time development of the right edge of the micropillar.** Distance from the reference line with the corresponding height as a function of time indicated by the colour for the micropillar shown in Fig. 1b. The purple and pink lines indicate the pillar shape before and after the stress drop investigated in Figs. 1c-e, respectively. The light gray horizontal line highlights the place where slip occurred.

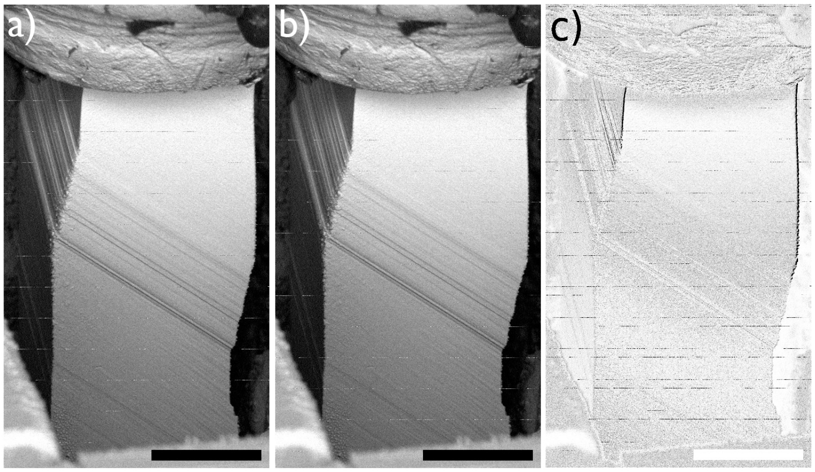


Supplementary Figure 9: **Locating spatial distribution of a strain burst.** **a**, **b**, Backscattered electron images of the micropillar before and after the stress drop analysed in Figs. 1c-e. The scale bar represents 20 µm. **c**, The difference of panels a) and b). The dark edges at the upper part of the pillar are due to plastic slip that occurred on the slip band highlighted in red in Fig. 1b.

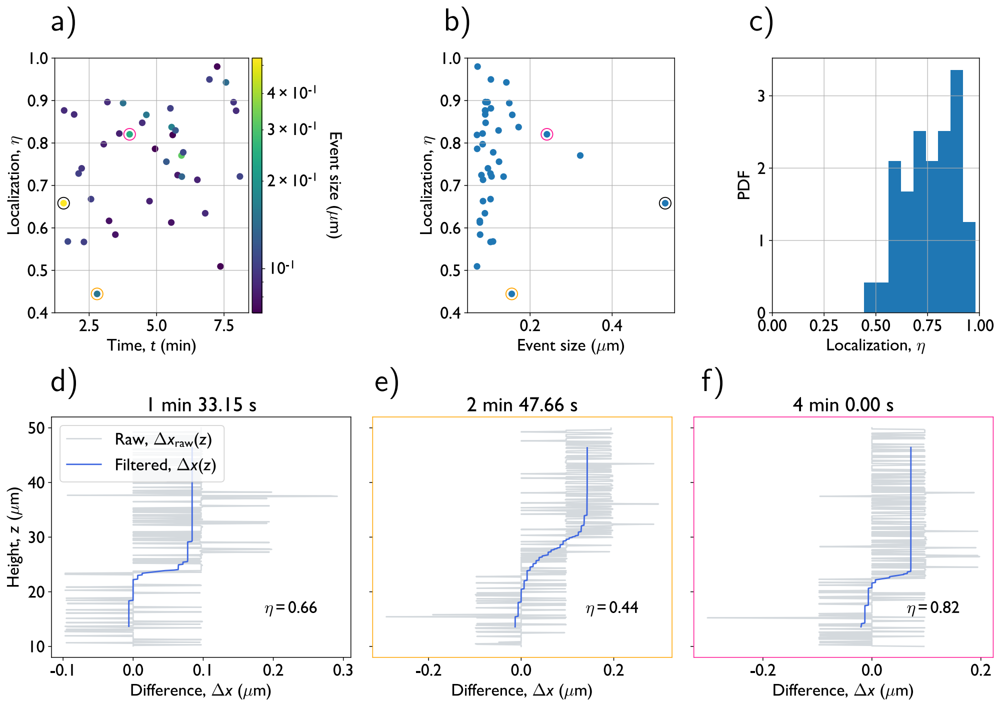


Supplementary Figure 10: **Analysis of strain localization on differential edge profiles.** **a**, Localization parameter $\eta$ as a function of time $t$ and event size $\Delta x(L) - \Delta x(0)$. The color scale refers to the event size $\Delta x(L) - \Delta x(0)$. **b**, Localization parameter $\eta$ as a function of event size $\Delta x(L) - \Delta x(0)$. **c**, The probabilty distribution of the localization parameter $\eta$. **d-f**, Three exemplary $\Delta x_{\text{raw}}(z)$ (light gray) and the corresponding $\Delta x(z)$ (blue) profiles obtained using the method described in the text. The datapoints corresponding to the curves are circled with the same colour as the frame of the figures.

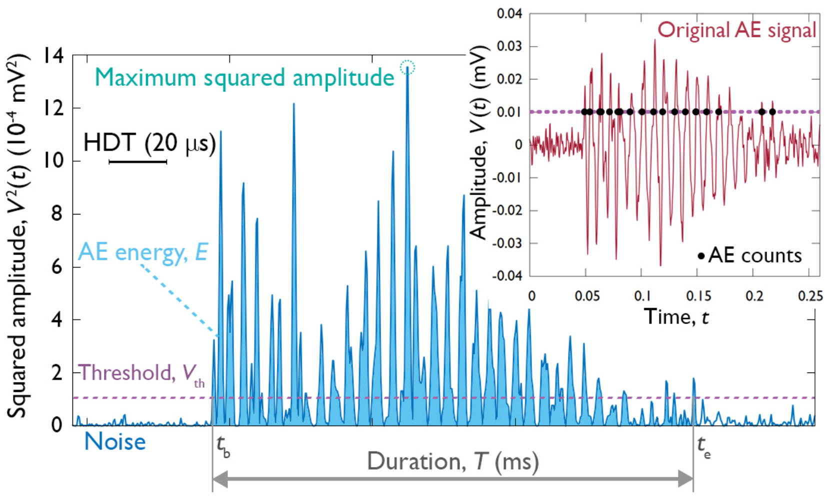


Supplementary Figure 11: **Parameters of a typical AE event.** Squared amplitude $V^2(t)$ of the AE signal as a function of time, showing the definitions of the AE parameters. The energy is the area of the region shaded in light blue. The inset presents the original waveform.

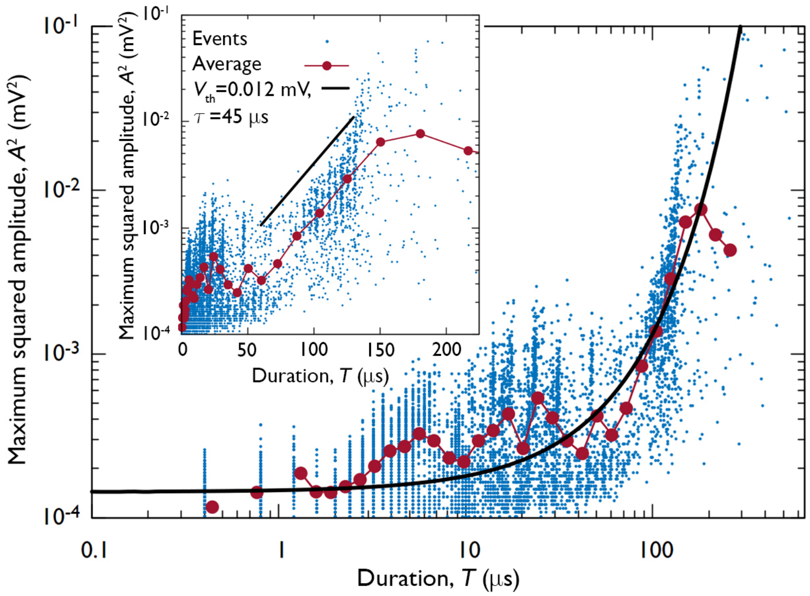


Supplementary Figure 12: **Analysis of AE signal attenuation.** Scatter plot of the maximum squared amplitude of individual AE events and their duration. The red data points represent the average relationship obtained by logarithmic binning with respect to the signal duration. Black solid line corresponds to the fit according to Eq. (8).

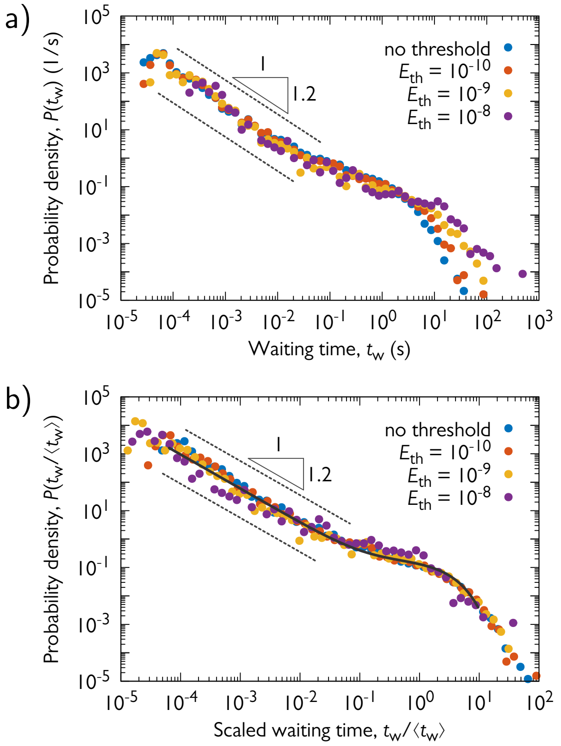


Supplementary Figure 13: **Effect of thresholding on waiting time distributions of $d = 32$ µm micropillars.** **a**, Waiting time distributions for AE events with energies larger than $E_{\text{th}}$. **b**, Distributions of panel a) re-scaled with the average waiting time of the events. The master curve fitting the collapsed curves is identical to that of Fig. 3f.

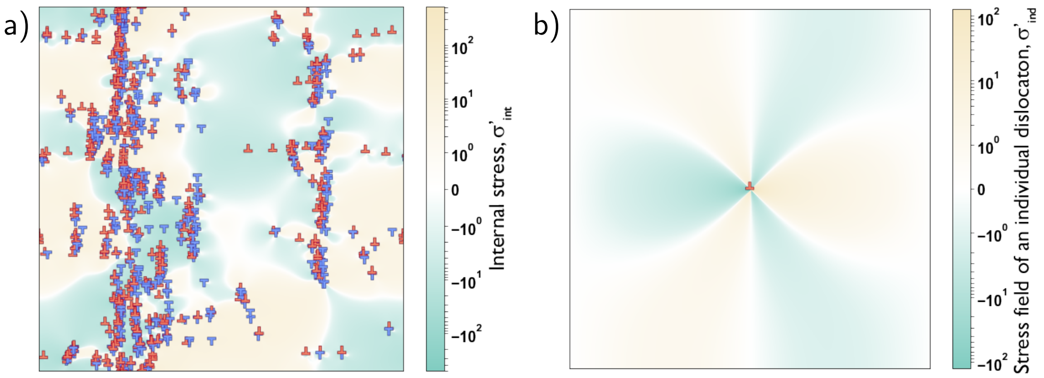


Supplementary Figure 14: **2D discrete dislocation dynamics simulations.** **a**, An exemplary configuration with 512 positive (red) and 512 negative (blue) sign dislocations. The background colour and the colour scale refers to the internal shear stress $\sigma'_{\mathrm{int}}$ generated by the individual dislocations defined as $\sigma'_{\mathrm{int}}(\mathbf{r}) = \sum_{i=1}^{N} s_i \sigma'_{\mathrm{ind}}(\mathbf{r} - \mathbf{r}_i)$. **b**, Shear stress field of an individual positive sign dislocation $\sigma'_{\mathrm{ind}}$ with periodic boundary conditions applied at all edges of the square-shaped simulation area.

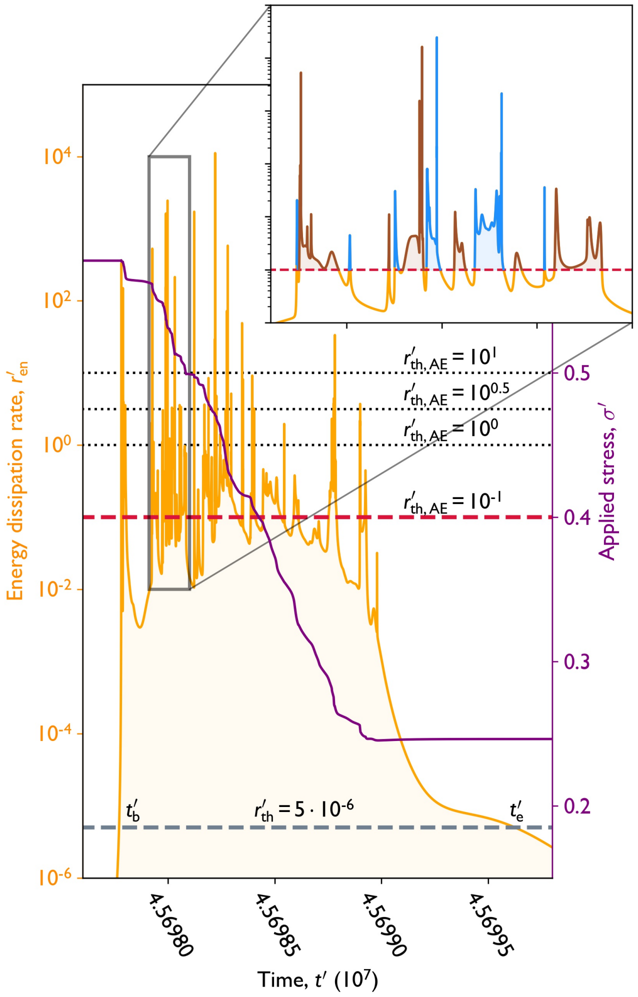


Supplementary Figure 15: **Event individualization in DDD simulations.** The time dependence of the energy dissipation rate $r'_{\mathrm{en}}$ during an exemplary plastic event (also shown in Supplementary Movie 3). The thick horizontal black line denotes the threshold $r'_{\mathrm{th}}$ used for identification of a plastic event, whereas dotted horizontal black lines refer to thresholds $r'_{\mathrm{th,AE}}$ used to individualize emulated AE bursts. The inset shows the identified AE bursts that took place during the stress drop at $r'_{\mathrm{th,AE}} = 0.1$ (shown with dotted red line in the main panel). The areas shaded alternately in blue and red correspond to the energies of the emulated AE events.

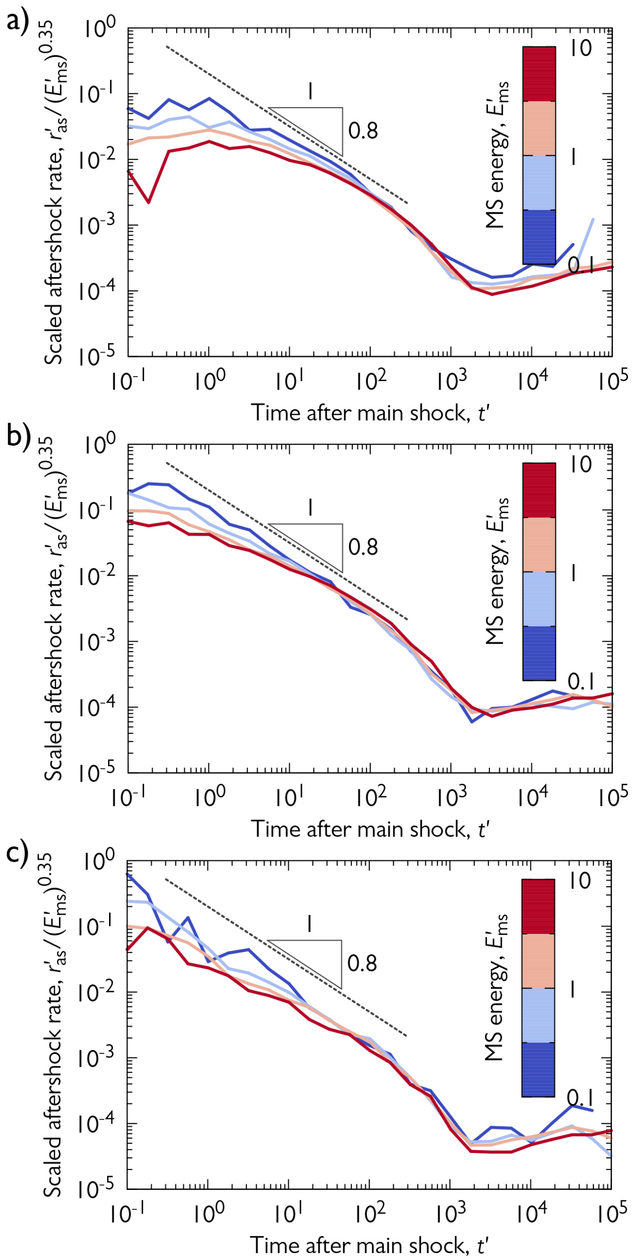


Supplementary Figure 16: **Effect of thresholding on the emulated scaled aftershock rates in DDD simulations.** Aftershock rates $r'_{as}$ after main shocks with different energies $E'_{ms}$ scaled with $(E'_{ms})^{0.35}$. The panels correspond to rates observed at different thresholds $r'_{th,AE}$ used for the emulation of AE events: **a**, $r'_{th,AE} = 0.1$. **b**, $r'_{th,AE} = 1$. **c**, $r'_{th,AE} = 10$.

## Supplementary Tables

| Quantity | length | stress | strain | time |
|---|---|---|---|---|
| Unit | $\rho^{-0.5}$ | $Gb\rho^{0.5}$ | $b\rho^{0.5}$ | $(Gb^2M\rho)^{-1}$ |

Supplementary Table 1: **Units of the dimensionless quantities used in the simulations.**

| Property | Earthquakes | Dislocation avalanches |
|---|---|---|
| Mechanism | Slip / crack | Dislocation movement |
| Expanse | in plane | in plane |
| Typical amplitude | m | nm |
| Typical reach | km | µm |
| Typical duration | minute − month | ms − s |
| Typical frequency | Hz | MHz |
| Size distribution | Gutenberg-Richter | Gutenberg-Richter |
| Aftershocks | Omori- and productivity law | Omori- and productivity law |

Supplementary Table 2: **Comparison between earthquake and dislocation avalanche properties.**
