# Supplementary material for: Dislocation avalanches are like earthquakes on the micron scale
Source: Nat Commun. 2022 Apr 13;13:1975. doi: 10.1038/s41467-022-29044-7 (PMC9007997; doi:10.1038/s41467-022-29044-7)
Supplement: Supplementary file 3 — Description of Additional Supplementary Files [file 41467_2022_29044_MOESM3_ESM.pdf]

Description of Additional Supplementary Files

File Name: Supplementary Movie 1
Description: In situ SEM video of a compression of a d=8 μm micropillar together with the measured force and the rate of AE events and released AE energies. The ultrasonic AE signal recorded during the compression was transformed into audible frequency domain that appears as a crackling noise.

File Name: Supplementary Movie 2
Description: Representative DDD simulation of N=1024 dislocations subjected to increasing shear stress with the protocol described in Methods. Dislocation configuration is seen in top right panel. Red and blue colours refer to the sign of the dislocations and the background colour with the colour scale represents the internal shear stress generated by the dislocations. The force-time curve is shown in the left panel together with the emulated AE count rate (see Methods for details).

File Name: Supplementary Movie 3

Description: Slowed down video of a representative plastic event (stress drop) from Supplementary Movie 2.

File Name: Supplementary Movie 4
Description: Edge detection during the deformation of a d=32 μm micropillar. Left panel shows the original video recorded by the SEM. In the left panel the green line is the reference (see Methods for details) and the blue line is the detected edge of the micropillar.
